# Infection…what else? The usefulness of procalcitonin in children after cardiac surgery

**DOI:** 10.1371/journal.pone.0254757

**Published:** 2021-10-22

**Authors:** Sara Bobillo-Perez, Monica Girona-Alarcon, Anna Sole-Ribalta, Carmina Guitart, Aida Felipe, Lluisa Hernandez, Monica Balaguer, Francisco Jose Cambra, Iolanda Jordan

**Affiliations:** 1 Disorders of Immunity and Respiration of the Pediatric Critical Patients Research Group, Institut Recerca Hospital Sant Joan de Déu, Universitat de Barcelona, Barcelona, Spain; 2 Pediatric Intensive Care Unit, Hospital Sant Joan de Déu, Universitat de Barcelona, Barcelona, Spain; 3 Pediatric Infectious Diseases Research Group, Institut Recerca Hospital Sant Joan de Déu, CIBERESP, Barcelona, Spain; University of Cape Town, SOUTH AFRICA

## Abstract

**Objectives:**

Procalcitonin is a useful biomarker for predicting bacterial infection after cardiac surgery. However, sometimes procalcitonin rises following cardiac surgery without a confirmation of bacterial infection. The aim was to analyse procalcitonin levels in children without a bacterial infection after cardiac surgery.

**Study design:**

This is a prospective, observational study of children <18 years old admitted to the pediatric intensive care unit after cardiac surgery.

**Results:**

1,042 children were included, 996 (95.6%) without a bacterial infection. From them, severe complications occurred in 132 patients (13.3%). Procalcitonin increased differentially depending on the type of complication. Patients who presented a poor outcome (n = 26, 2.6%) had higher procalcitonin values in the postoperative period than the rest of patients (<24 hours: 5.8 ng/mL vs. 0.6 ng/mL; 24–48 hours, 5.1 ng/mL vs. 0.8 ng/mL, and 48–72 hours, 5.3 ng/mL vs. 1.2 ng/mL), but these values remained stable over time (p = 0.732; p = 0.110). The AUC for procalcitonin for predicting poor outcome was 0.876 in the first 24 hours. The cut-off point to predict poor outcome was 2 ng/mL in the first 24 hours (sensitivity 86.9%, specificity 77.3%). Patients with bacterial infection (n = 46) presented higher values of procalcitonin initially, but they decreased in the 48–72 hours period (<24 hours: 4.9 ng/mL; 24–48 hours, 5.8 ng/mL, and 48–72 hours, 4.5 ng/mL).

**Conclusions:**

A procalcitonin value<2 ng/mL may indicate the absence of infection and poor outcome after cardiac surgery. The evolution of the values of this biomarker might help to discern between infection (where procalcitonin will decrease) and poor outcome (where procalcitonin will not decrease).

## Introduction

Congenital heart disease is the most frequent malformation in newborns [1]. Many cases of congenital heart disease require cardiac surgery during childhood, which is a complex procedure that usually requires extracorporeal circulation. Thanks to the improvements in surgical techniques, in the protective measures implemented during procedures, and in postoperative care, mortality rates have decreased significantly in the last twenty years [2–4]. Therefore, current concerns are focused on reducing the morbidity associated with cardiac surgery, especially neurological injuries related to surgery [5–7].

A systemic inflammatory reaction can be triggered by a surgical procedure [8], especially complex surgeries with prolonged extracorporeal circulation, aortic cross-clamping, and deep hypothermic circulatory arrest [9, 10]. The clinical distinction between bacterial infection and systemic inflammatory response syndrome can be difficult to make in children, particularly in those who are younger and in newborns. Starting 10 years ago, procalcitonin (PCT) has been used to differentiate these two entities [11, 12]. This 116-aminoacid protein is practically undetectable in healthy children [13]. However, PCT levels increase in the presence of bacterial endotoxins and their lipopolysaccharides, which act like a trigger for its secretion, and are also mediated by tumor necrosis factor-alpha, interleukin-1 beta, and interleukin-6 [13]. PCT response is fast and runs in parallel to the severity of the bacterial infection, and this biomarker has been demonstrated to be superior for detecting invasive infection in these patients in comparison with other biomarkers like C-reactive protein, white blood cells count, or pro-adrenomedullin [14]. PCT also facilitates monitoring the evolution of an infection due to its rapid decline once the infection is brought under control. Secondary to this intrinsic feature, it has been proposed as a tool for antibiotic stewardship in children with a suspected bacterial infection after cardiac surgery [15, 16].

However, PCT rises in some children following cardiac surgery without a confirmation of bacterial infection [17], so we hypothesized that other postoperative factors might determine an increase in the levels of PCT after a cardiac surgery. Some important complications can arise and make the postoperative period difficult, such as low cardiac output syndrome (LCOS) [18], severe pulmonary hypertension, and arrhythmia. A relationship between PCT and organ dysfunction has been reported [19, 20], and PCT might increase after cardiac surgery due to the organ dysfunction.

The aim of this study was to analyze PCT after cardiac surgery in those patients without confirmed infection during the postoperative period and to detect the potential value of PCT in diagnosing postoperative complications. The secondary aim was to compare PCT in infected patients and patients with poor outcomes.

## Methods

This was a prospective unicenter observational study performed in the pediatric intensive care unit (PICU) of a pediatric tertiary referral hospital between January 2012 and December 2019. Children recovering from cardiac surgery were included. The exclusion criteria were: presence of a confirmed community-acquired infection before surgery, newborns who were admitted to the neonatal intensive care unit after cardiac surgery (which is a different unit), and patients with rheumatic diseases (PCT values can be altered due to their condition). The study was conducted in accordance with the Declaration of Helsinki recommendations and was approved by the local Ethical Assistance Committee and the Institutional Review Board (CEIm Fundació Sant Joan de Déu, Barcelona. 2^nd^ February 2012, and code PIC-66-11) with the waiver of the parental informed consent.

The primary objective was to analyze PCT levels in those patients without an invasive bacterial infection after cardiac surgery and to explore the possible relationship of PCT and complications in the postoperative period.

Secondary objective was to compare the evolution of PCT over time between patients with poor outcome and patients with an invasive bacterial infection.

According to our local protocol, PCT assessment was carried out daily from the moment of admission to the PICU after cardiac surgery until the transfer of the patient to the ward. PCT values were determined by LumiTest PCT immunoluminometric assay (ATOM SA; Brahms Diagnostica), which uses two monoclonal antibodies and requires 20 μL of serum or plasma. A PCT value of <0.5 ng/mL was considered normal.

Baseline demographic data was recorded and included: age, gender, type of congenital heart disease, complexity of surgery (STAT Mortality Categories) [21], surgery times (duration of extracorporeal circulation, aortic cross-clamping and deep hypothermic circulatory arrest), and Pediatric Risk of Mortality Score (PRISM III). The hemodynamic and respiratory support required in the first 3 days after surgery was analyzed using the Vasoactive-Inotropic Score (VIS), along with the duration of the mechanical ventilation. In our unit, milrinone is administered during the first 24 hours after extracorporeal circulation by protocol. The diagnosis of a hospital-acquired invasive bacterial infection was defined according to Center for Disease Control criteria [22]. Only confirmed bacterial infections (positive culture) were considered, including ventilator-associated pneumonia (VAP), central line-associated bloodstream infection (CLABSI), catheter-associated urinary tract infection, and surgical wound infections.

Outcomes were considered as presence of complications, length of stay (LOS) in the PICU and in the hospital, and death. The complications considered were: need for unplanned reintervention or cardiac catheterization, arrhythmias, LCOS (ventricular dysfunction determined by echocardiopraphy with a left ventricular ejection fraction under 40%), cardiac arrest, need for renal replacement therapy, and need for extracorporeal support in the postoperative period. The need for renal replacement therapy or extracorporeal support, cardiac arrest, or death during the stay in the PICU were considered poor outcomes [23].

SPSS 26.0 for Windows was used for statistical analysis. Categorical variables were expressed as frequencies and percentages and compared using Pearson’s chi-square test. Continuous variables were expressed as median and interquartile range (IQR) or mean and standard deviation, and compared using the Mann-Whitney U test or t-student (used for paired samples). Napierian logarithm (NL) was applied to the PCT values to achieve a normal distribution. For multiple comparisons between the different subgroups (regarding the presence or absence of infection and/or poor outcome), one-way ANOVA with Bonferroni correction were applied. The discriminatory power was evaluated with receiver operating characteristic curves and area under the curve (AUC). Sensitivity (Sn), specificity (Sp), and the predictive values were evaluated using MedCalc 13.0 for Windows. Multivariable logistic regression was used to evaluate the independent influence of different variables as regards the PCT cut-off. Variables with p < 0.1 in the univariable analysis were considered for inclusion in the logistic regression model. The final model was determined using forward stepwise selection, with significance level of 0.1 for entry in the model. These results were expressed as odds ratio (OR) and 95% confidence interval (CI), and represented in a forest plot. A p-value of <0.05 was considered significant.

## Results

**Fig 1:**
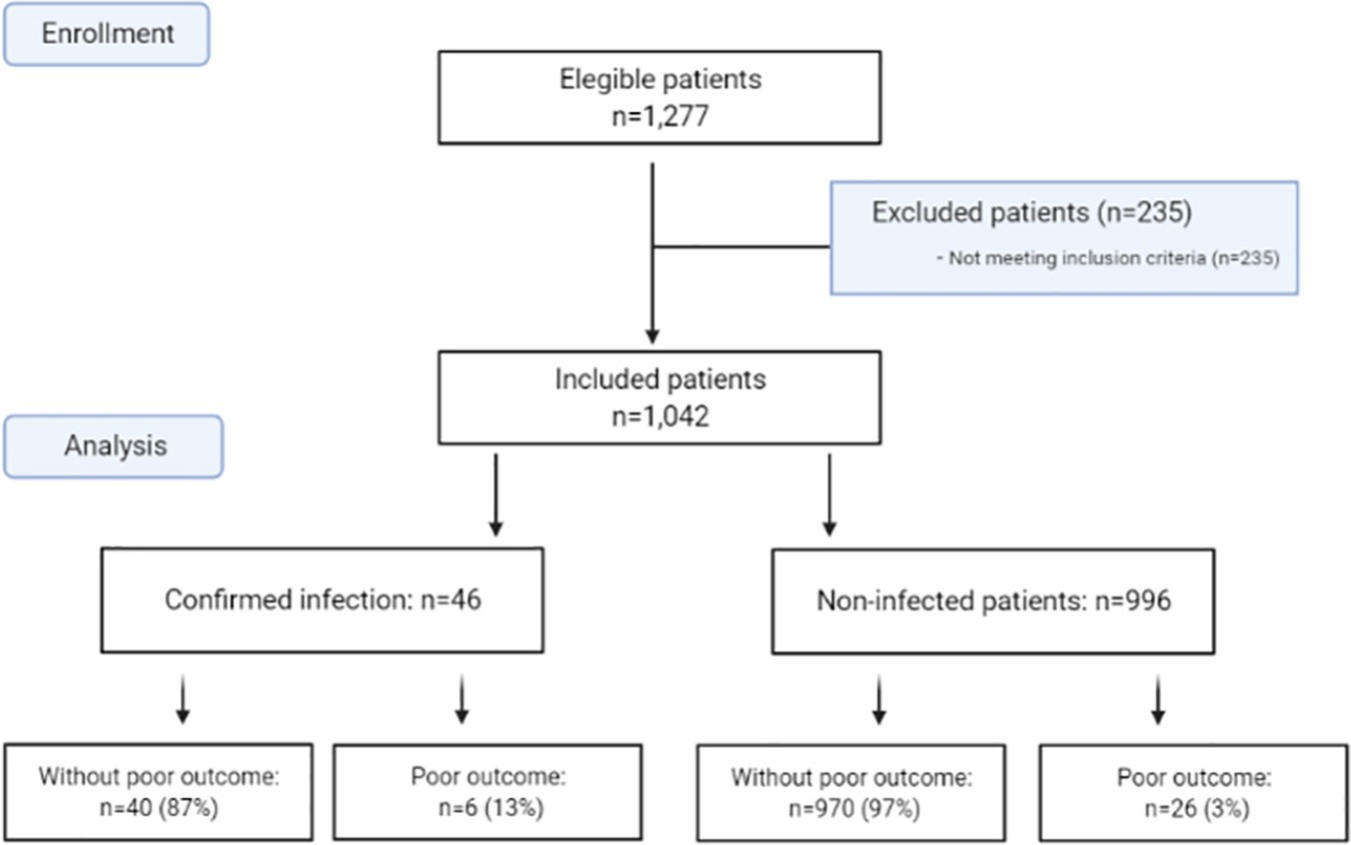
Flow chart for patients. Excluded patients were those who spend the postoperative period in the Neonatal Intensive Care Unit.

During the study period, 1,042 children were included. Forty-six had a confirmed bacterial infection in the postoperative period. The flow chart for patients is represented in Fig 1. Table 1 compares patients regarding the presence or absence of a bacterial infection during the postoperative period.

**Table 1:** General description of the sample including demographic, surgical and clinical data, and comparison between patients with and without bacterial infection after surgery.

Variables	TOTAL	Absence of infection	Presence of infection	p-value
	n = 1,042	n = 996	n = 46	
Male	571 (54.8)	545 (54.7)	26 (56.5)	0.810
Age (years)	1.6 (0.5–6.2)	1.8 (0.6–6.4)	0.5 (0.2–1.3)	<0.001
Weight (kg)	10 (6–20)	10 (6.3–20)	6 (4.4–9.1)	<0.001
STAT>3	58 (5.6)	51 (5.1)	7 (15.2)	0.004
Previous surgery	342 (32.8)	326 (32.7)	16 (34.8)	0.772
Syndrome	156 (15.0)	144 (14.5)	12 (26.1)	0.031
EC time (min)	70 (48–98)	70 (48–96)	87 (66–144)	0.001
Cross-clamp time (min)	39 (25–61)	38 (24–60)	49.5 (34–106)	0.001
DHCA time (min)	28.5 (22–39)	28.5 (22–38)	34 (14–45)	0.671
PRISM	3 (2–6)	3 (2–6)	7 (3–11)	<0.001
Mechanical ventilation (hours)	6 (3–24)	5.5 (3–24)	84 (24–216)	<0.001
VIS 24 hours (points)	3.7 (3.7–7)	3.7 (3.7–7)	8.9 (3.7–18.7)	<0.001
VIS 48 hours (points)	0 (0–3.7)	0 (0–3.7)	7.9 (3.7–13.7)	<0.001
VIS 72 hours (points)	0 (0–0)	0 (0–0)	3.7 (0–8.8)	<0.001
Reintervention/cath	33 (3.2)	28 (2.8)	5 (10.9)	0.002
Severe pulmonary hypertension	24 (2.3)	19 (1.9)	5 (10.9)	0.003
Arrhythmia	72 (6.9)	67 (6.7)	5 (10.9)	0.241
LCOS	55 (5.3)	60 (5)	5 (10.9)	0.090
Cardiopulmonary arrest	14 (1.3)	10 (1)	4 (8.7)	0.002
RRT	18 (1.7)	15 (1.5)	3 (6.5)	0.041
ECMO	10 (1)	8 (0.8)	2 (4.3)	0.068
Poor outcome	32 (3.1)	26 (2.6)	6 (13)	<0.001
Death in the PICU	13 (1.2)	9 (0.9)	4 (8.7)	0.002
LOS in the PICU	3 (2–5)	3 (2–5)	6.5 (4–14)	<0.001
LOS in the hospital	7 (6–10)	7 (6–9)	12.5 (8–23)	<0.001

Continuous values expressed as median (IQR). Comparison made using Mann-Whitney U test. DHCA: deep hypotermic circulatory arrest. LCOS: low cardiac output syndrome. LOS: length of stay. ECMO: extracorporeal membrane oxygenation. RRT: renal replacement therapy. PICU: pediatric intensive care unit. VIS: vasoactive inotropic score.

### Analysis of the patients without an invasive bacterial infection

The median age was 1.8 years (IQR 0.6–6.4), and 545 were males (54.7%). Tetralogy of Fallot (161, 16.2%), and interventricular communication (149, 15%) were the most frequent congenital heart disease. The most frequent STAT Mortality Category was 2, with 431 (43.3%) children. One hundred sixty-one patients (16.2%) had an STAT Mortality Category superior to 3. A total of 593 patients (59.5%) were admitted without invasive mechanical ventilation and inotropic support was not required in 679 (68.2%). Severe complications occurred in 132 patients (13.3%). Table 1 includes the main description of these complications. The most frequent ones were arrhythmia (67, 6.7%), followed by LCOS (50, 5%). A total of 26 patients (2.6%) had a poor outcome and 9 (0.9%) died.

**Fig 2:**
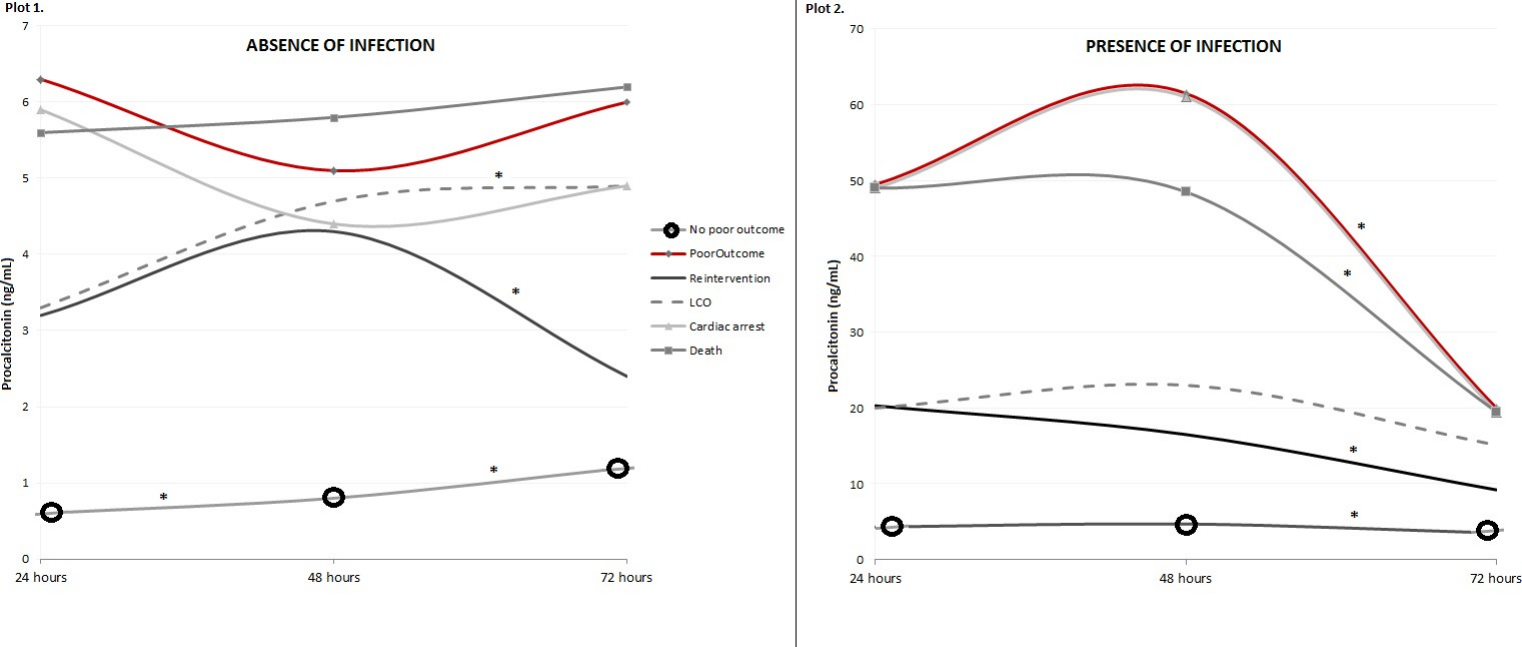
Representation of the median values of procalcitonin (PCT) as regards the different complications within 24, 48 and 72 hours of the cardiac surgery. Values of PCT expressed as median (IQR) in ng/mL. Plot 1 represents patients without bacterial infection and PCT values range between 0 and 7 ng/mL. Plot 2 represents patients with bacterial infection and PCT values range between 0 and 70 ng/mL. Comparisons over time were performed with paired samples t-student test considering the transformation by Napierian logarithm of the PCT values. * marks the statistically significant differences (p-value <0.05). LCO: Low cardiac output.

In the absence of severe complications during the postoperative period, PCT values remained low, with a median of 0.54 ng/mL (IQR 0.2–1.6) during the first 24 hours and 0.8 ng/mL (IQR 0.3–2.2) between 48 and 72 hours. However, in the presence of complications, PCT increased differentially depending on the type of complication. These different values are summarized in Fig 2. There were no statistically significant differences regarding each complication and the evolution of PCT over time in the absence of bacterial infection (PCT during the first 24 hours vs. 24–48 hours and 24–48 hours vs. 48–72 hours), with two exceptions: with ventricular dysfunction, it increased in the 24–48 hours period (p<0.001) and in unplanned re-interventions, it increased during the same period (p = 0.026) but then decreased significantly between 48 and 72 hours (p = 0.019). Both are represented in Fig 2.

Table 2 compares the different values of PCT according to the presence or absence of each complication. Furthermore, statistically significant differences were detected regarding each complication and PCT in the first 72 hours after surgery (except severe pulmonary hypertension and PCT at 72 hours). Patients who presented a poor outcome had higher PCT values in the postoperative period than the rest of patients: within 24 hours, 5.8 ng/mL (IQR 2.7–34.7) vs. 0.6 ng/mL (IQR 0.2–1.8); between 24 and 48 hours, 5.1 ng/mL (IQR 2.2–39.4) vs. 0.8 ng/mL (IQR 0.4–2.6); and between 48 and 72 hours, 5.3 ng/mL (IQR 1.9–50.4) vs. 1.2 ng/mL (IQR 0.4–3.6), respectively, all with p<0.001). However, these values remained stable over time (Comparisons of NL PCT with respect to time periods: 24 vs. 48 hours, p = 0.732, and 48 vs. 72 hours p = 0.110).

**Table 2:** Comparison of procalcitonin (ng/mL) values in the presence or absence of different complications.

Complication	State	Procalcitonin	Procalcitonin	Procalcitonin
		24 hours	48 hours	72 hours
LCOS	*Yes*	3.3 (0.9–11.2)	4.7 (1.0–12.2)	4.9 (1–10.4)
	*No*	0.6 (0.2–1.8)	0.8 (0.3–2.4)	1.15 (0.4–3)
	p-value	<0.001	<0.001	<0.001
Re-intervention /cath lab	*Yes*	3.2 (1–14)	4.3 (0.9–43.9)	2.7 (0.7–20)
	*No*	0.6 (0.2–1.9)	0.85 (0.4–2.6)	1.2 (0.4–3.7)
	p-value	<0.001	<0.001	0.013
Severe pulmonary hypertension	*Yes*	2.3 (0.5–6.9)	2.8 (0.7–5.8)	2 (0.6–4.9)
	*No*	0.6 (0.2–2)	0.9 (0.4–2.6)	1.2 (0.5–4)
	p-value	0.015	0.039	0.509
Renal replacement therapy	*Yes*	12.5 (2.9–42.5)	14.6 (2.2–96.5)	13.2 (1.2–76.7)
	*No*	0.6 (0.2–1.9)	0.8 (0.4–2.6)	1.2 (0.4–3.7)
	p-value	<0.001	<0.001	<0.001
ECMO	*Yes*	22 (6–35)	13 (4.5–107)	9 (2.7–109)
	*No*	0.6 (0.21–2)	0.9 (0.4–2.6)	1.2 (0.5–4)
	p-value	<0.001	<0.001	0.001
Death in the PICU	*Yes*	5.6 (2.3–22)	5.8 (2.1–12.5)	6.2 (4.2–36.3)
	*No*	0.6 (0.2–2)	0.9 (0.4–2.6)	1.2 (0.5–4)
	p-value	<0.001	<0.001	0.001

Values expressed as median (IQR). Comparison made using Mann-Whitney U test. LCOS: low cardiac output syndrome. ECMO: extracorporeal membrane oxygenation. PICU: pediatric intensive care unit.

The discriminatory capacity of PCT was assessed and the AUC for predicting poor outcome was 0.876 (95% CI 0.851–0.899) in the first 24 hours and 0.823 (95% CI 0.745–0.900) between 24 and 48 hours. A PCT of >2 ng/mL in the first 24 hours was chosen as a marker for poor outcome and its Sn, Sp, and predictive values are included in Table 3. In addition, Table 3 includes the predictive values for this cut off point when considering its ability to discriminate complications and LCOS.

**Table 3:** Predictive ability of the cut off point (procalcitonin > 2 ng/mL during the first 24 hours) and the different outcomes.

Variable	Sn	Sp	PPV	NPV
Poor outcome	86.9 (66.4–97.2)	77.3 (74.1–80.3)	10.6 (6.6–16)	99.5 (98.5–99.9)
Complications	45.4 (36.2–54.8)	79.2 (75.9–82.3)	28.7 (22.4–35.8)	88.7 (85.8–91.2)
LCOS	58 (43.2–71.8)	77.7 (74.5–80.7)	15.4 (10.6–21.4)	96.3 (94.5–97.7)

Values expressed as % (95% confidence interval). Sn = Sensitivity, Sp = Specificity, PPV = Positive predictive value, NPV = Negative predictive value.

It was decided to divide the patients into two groups according to the defined cut-off point and assess the presence of complications based on this division. A PCT level up to 2 ng/mL was detected during the postoperative period in 249 patients (25%). Table 4 compares the differences between patients with a PCT of <2 ng/mL and patients with a PCT of >2 ng/mL.

**Table 4:** Differences between patients in their procalcitonin (PCT) values in the first 24 hours, and the main data, including the support required in the intensive care unit, and outcomes.

Variables	PCT<2 ng/ml (n = 746)		PCT>2ng/ml (n = 249)		p-value
Male	386	51.7%	159	63.9%	0.001
Age (years)	1.8	(0.6–6.5)	1.8	(0.5–6.35)	0.554
Weight (kg)	10	(6.4–20)	10	(6–20)	0.352
STAT>3	108	14.5%	53	21.3%	0.012
Previous surgery	214	28.7%	112	45.0%	<0.001
Syndrome	105	14.1%	39	15.7%	0.597
EC time (min)	65	(46–90)	87	(59–122.5)	<0.001
Cross-clamp time (min)	35.5	(23–57)	47.5	(27–74)	<0.001
DHCA time (min)	27	(23.25–36.75)	32	(18.75–38.25)	1.000
PRISM	3	(2–5)	5	(2–8)	<0.001
MV (hours)	4	(3–14.25)	18	(4–72)	<0.001
VIS 24 hours (points)	3.7	(3.7–3.7)	5	(3.7–13.15)	<0.001
VIS 48 hours (points)	0	(0–3.7)	3.7	(0–8.7)	<0.001
VIS 72 hours (points)	0	(0–0)	0	(0–3.7)	<0.001
Complications	65	8.7%	67	26.9%	<0.001
Reintervention/cath	9	1.2%	19	7.6%	0.023
Severe PH	10	1.3%	9	3.6%	<0.001
Arrhythmia	36	4.8%	31	12.4%	<0.001
LCOS	16	2.1%	34	13.7%	<0.001
Cardiopulmonary arrest	1	0.1%	9	3.6%	<0.001
RRT	2	0.3%	13	5.2%	<0.001
ECMO	1	0.1%	7	2.8%	<0.001
Poor outcome	3	0.4%	23	9.2%	<0.001
Death	1	0.1%	8	3.2%	<0.001
LOS in the PICU	3	(2–4)	5	(3–8)	<0.001
LOS in the hospital	7	(6–8)	9	(7–16)	0.001

DHCA: Deep hypotermic circulatory arrest; ECMO: Extracorporeal membrane oxygenation; LCOS: low cardiac output syndrome; LOS: length of stay; MV: Mechanical ventilation; PH: pulmonary hypertension; PICU: Pediatric intensive care unit; RRT: Renal replacement therapy. Categorical variables expressed as frequencies (percentages), compared with Chi-square test. Continuous variables expressed as median (IQR) and compared with Mann-Whitney U test.

In patients with increased PCT, the frequency of complications during the postoperative period was higher, with statistically significant differences with respect to patients with a PCT of <2 ng/mL. The greatest differences were detected in patients with LCOS. Of the 26 patients who presented poor outcome, a PCT of > 2ng/mL was detected in 23 (88.4%) during the postoperative period.

**Fig 3:**
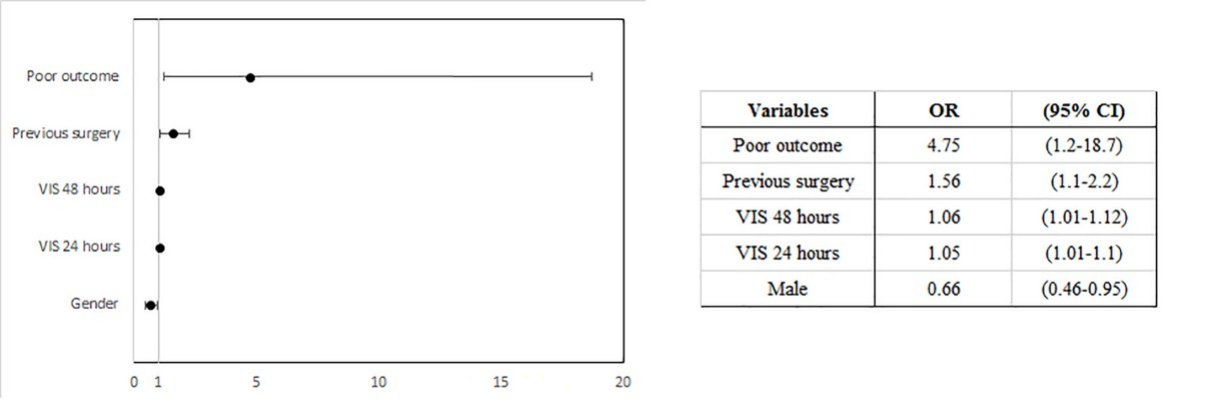
Forest plot representing the results of the logistic regression. Variables included in the multivariable model: gender, STAT>3, previous surgery, surgery times, VIS at 24 and 48 hours, low cardiac output syndrome, poor outcome. OR: odds ratio, CI: confidence interval.

In the multivariable analysis, the poor outcome showed the greater independent association with a PCT higher than 2 ng/mL in the first 24 hours: OR 4.75 (95% CI 1.2–18.7). Fig 3 represents the different variables included in the multivariable model.

### Comparison between patients with poor outcome and patients with an invasive bacterial infection

**Fig 4:**
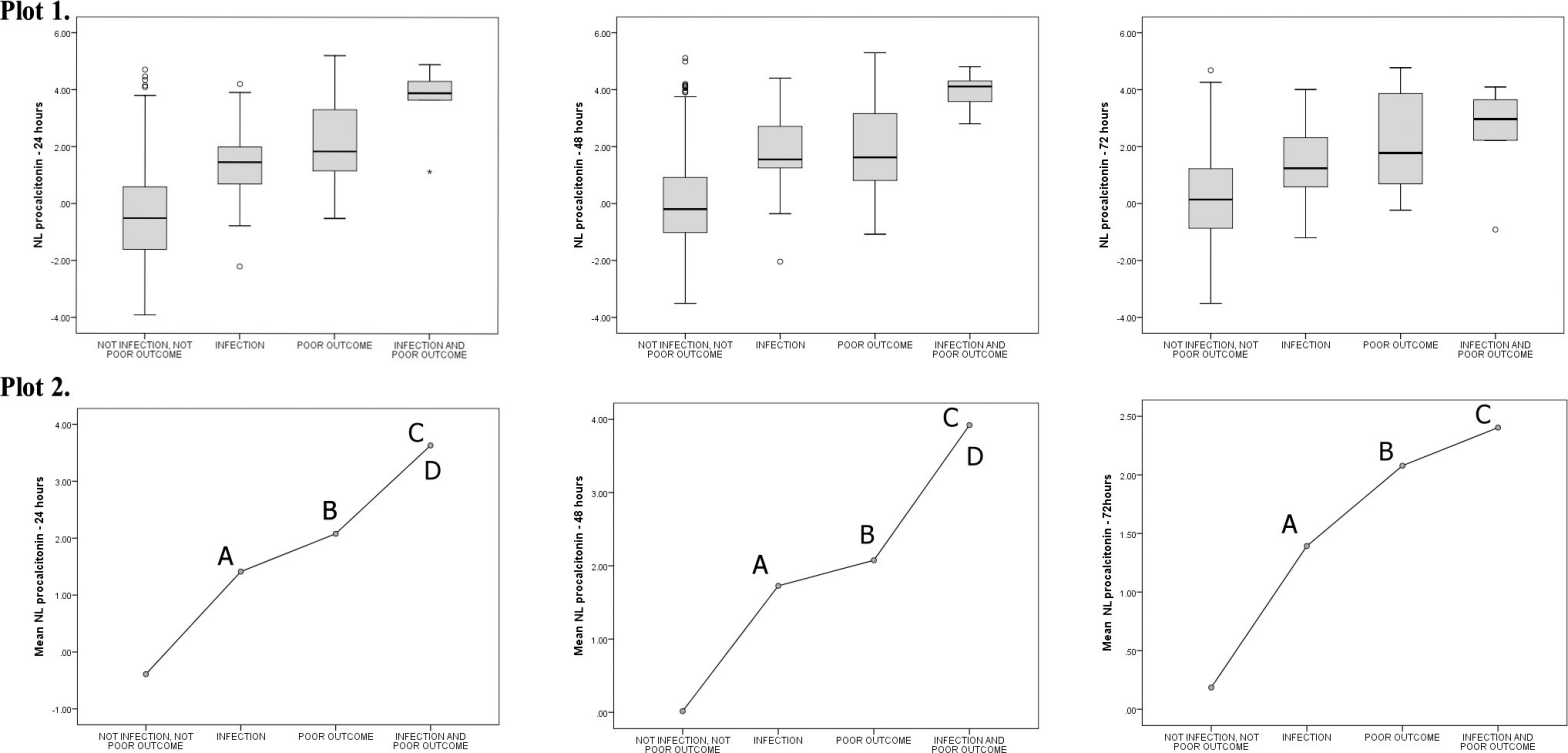
Different levels of procalcitonin with respect to the presence of infection and/or poor outcome. Procalcitonin expressed as the Napierian logarithm (NL) of procalcitonin and compared using the ANOVA test. Plot 1 represents the different NL procalcitonin values for each category in the 3 time periods. Plot 2 includes the comparison between the different clinical situations, regarding the presence of infection and poor outcome. Only the statistically significant differences (p<0.050) are marked. Comparisons legend: A) Not infection, not poor outcome vs. infection; B) Not infection, not poor outcome vs. poor outcome; C) Not infection, not poor outcome vs. infection and poor outcome; D) Infection vs. infection and poor outcome; E) Infection vs. poor outcome; F) Poor outcome vs. infection and poor outcome.

Considering the total of the sample (n = 1,042), 46 patients presented an invasive bacterial infection (4.4%). VAP was the most frequent bacterial infection (n = 31, 67.4%), followed by CLABSI (n = 8, 17.4%). These infected patients presented PCT values of 4.9 ng/mL (IQR 2.4–20) within 24 hours, 5.8 ng/mL (IQR 3.5–23) within 48 hours, and 4.5 ng/mL (IQR 1.8–13.7) within 72 hours, with a significant decrease between 48 and 72 hours (Comparisons of NL PCT with respect to time periods: 24 vs. 48 hours, p = 0.111; 48 vs. 72 hours, p<0.001). Fig 4 represents the different PCT values for infected patients without poor outcome, patients with poor outcome, and patients with both. The infected patients with poor outcome presented the higher values of PCT. Additionally, the values of PCT decreased after 48 hours in infected patients, while the values of PCT remained stable in patients with poor outcome. Table 5 compares the differences between patients with a PCT of <2 ng/mL and patients with a PCT of >2 ng/mL including all patients, regardless of the diagnosis of confirmed bacterial infection.

**Table 5:** Differences between patients regarding the values of procalcitonin (PCT) in the first 24 hours, and the main data and outcomes.

Variables	PCT<2 ng/ml (n = 751)		PCT>2 ng/ml (n = 291)		p-value
Male	389	51.8%	182	62.5%	0.002
Age (years)	1.8	(0.6–6.6)	1.2	(0.5–5.7)	0.036
Weight (kg)	10	(6.4–20)	9	(5.5–17)	0.018
STAT>3	109	14.6%	67	23%	0.001
Previous surgery	214	28.5%	128	44.0%	<0.001
Syndrome	105	14.0%	51	17.5%	0.247
EC time (min)	65	(46–90)	87	(60–123)	<0.001
Cross-clamp time (min)	35	(23–57)	49	(29–77)	<0.001
DHCA time (min)	27	(24.5–33.5)	32	(20–39)	0.887
PRISM	3	(2–5)	5	(3–9)	<0.001
MV (hours)	4	(3–15)	24	(4–96)	<0.001
VIS 24 hours (points)	3.7	(3.7–3.7)	6.5	(3.7–13.7)	<0.001
VIS 48 hours (points)	0	(0–3.7)	3.7	(0–10)	<0.001
Reintervention/cath	9	1.2%	24	8.2%	<0.001
Severe PH	10	1.3%	14	4.8%	0.001
Arrhythmia	36	4.8%	36	12.4%	<0.001
LCOS	16	2.1%	39	13.4%	<0.001
Cardiopulmonary arrest	1	0.1%	13	4.5%	<0.001
RRT	2	0.3%	16	5.5%	<0.001
ECMO	1	0.1%	9	3.1%	<0.001
Poor outcome	3	0.4%	29	10%	<0.001
Bacterial infection	5	0.7%	41	14.1%	<0.001
Death	1	0.1%	12	4.1%	<0.001
LOS in the PICU	3	(2–4)	5	(3–9)	<0.001
LOS in the hospital	7	(6–8)	9	(7–16)	<0.001

DHCA: Deep hypotermic circulatory arrest; ECMO: Extracorporeal membrane oxygenation; LCOS: low cardiac output syndrome; LOS: length of stay; MV: Mechanical ventilation; PH: pulmonary hypertension; PICU: Pediatric intensive care unit; RRT: Renal replacement therapy. Categorical variables expressed as frequencies (percentages), compared with Chi-square test. Continuous variables expressed as median (IQR) and compared with Mann-Whitney U test.

In the multivariable analysis, both poor outcome and infection showed the greater independent association with a PCT higher than 2 ng/mL in the first 24 hours: OR 11.22 (95% CI 3.2–39.9) and OR 24.03 (95% CI 9.2–62.5), respectively.

## Discussion

This is the first study that analyzes the relationship between PCT and severe complications after cardiac surgery in patients without bacterial infection. Our results underscore the high frequency of increased PCT in those patients who subsequently have poor outcomes. In addition, the evolution over time of PCT should help physicians to discriminate between bacterial infection and poor outcomes.

PCT is a widely accepted and useful biomarker for invasive bacterial infection in children. Its use has been extended to monitor sepsis [24, 25], respiratory tract infections [26], bronchiolitis [27], hospital-acquired infections [28], and also patients after cardiac surgery [29]. A value over 2 ng/mL within the first 24 hours of cardiac surgery with cardiopulmonary bypass is the recommended cut-off to alert physicians about the risk of an underlying invasive bacterial infection. Between 24 and 48 hours, the cut-off is established at over 4 ng/mL [11].

LCOS is the set of symptoms and signs derived from an alteration in the ability of the heart to ensure the correct delivery of oxygen and nutrients to the tissues [30]. The systolic volume decreases due to the impaired cardiac contractility, and the body tries to compensate for this reduction in the cardiac output by increasing the heart rate and releasing compensatory hormones. Other biomarkers besides PCT have been studied to determine their ability to detect LCOS in the postoperative period. In children, atrial natriuretic peptide, b-type natriuretic peptide, copeptin, pro-adrenomedullin, and cardiac troponin I have been analyzed by Perez-Navero *et al*. [18], and the combination of troponin I and pro-adrenomedullin was shown to have the greatest AUC for predicting LCOS. The proteins included in this analysis are released as compensatory agents or as markers of myocardial injury, and PCT was not included in this analysis. In comparison with other articles that reported a LCOS rate around 25% [18, 31], our results showed only 5%. However, our data evinced higher values of PCT in those patients with LCOS compared to those with normal/acceptable cardiac function in the postoperative period. The role of PCT may be related to the release of PCT linked to organ dysfunction and ischemia-reperfusion injury. Some circumstances promote the release of PCT in the absence of a bacterial trigger, and in most cases this is linked to a critical situation [32, 33]. For example, after cardiac arrest, patients present higher PCT values in the first 12 hours after the event [19], and these values progressively decrease if the clinical evolution is good, following a kinetic model like that reported for sepsis that responds well to antibiotics.

Furthermore, both populations, infected and non-infected patients, presented high values of PCT initially, but while PCT decreased between 24 and 48 hours in infected patients, in patients with poor outcomes it did not decrease. Remarkably, infected patients with poor outcomes had even higher values of PCT, and these still decreased during the 24–48 hours postoperative period. Indeed, the antibiotic treatment that controlled the bacterial infection determined a decrease in the levels of PCT in the 24–48 hours period, independently of the presence of poor outcomes.

The strength of our study was the inclusion of a large cohort to analyze, for the first time, the behavior of PCT in children without bacterial infection after cardiac surgery. A feature of PCT can be highlighted from our results: its power to identify those patients who will have a postoperative period without severe complications or poor outcomes. A value of PCT over 2 ng/mL showed a high NPV (88.7% for severe complications and 99.5% for poor outcomes, respectively), which suggests that if the PCT is less than 2 ng/mL, the presence of a poor outcome or complications would be unlikely. Conversely, a high PCT value in the postoperative period should alert physicians to a high risk of the patient developing or having a serious complication.

This study presents some limitations that should be stated. Firstly, it is a single-center design. However, the large sample of subjects included may counteract this limitation. Secondly, only confirmed bacterial infections (culture positive) were considered as hospital-acquired bacterial infections to facilitate future cross-site benchmarking, but we acknowledge that current culture techniques in children have low sensitivity and some probable/possible bacterial infections may have altered PCT values. Future research should explore the influence of probable bacterial infections on the ability of PCT to predict complications after cardiac surgery. Our results should be considered as preliminary due to the absence of similar studies in the bibliography. It would be interesting to delve deeper into the analysis of this biomarker in the absence of infection in order to provide information that has not been previously considered.

## Conclusions

All in all, an increased level of PCT in the postoperative period should be considered as an indicator for severe complication after cardiac surgery. Moreover, the evolution of the values of this biomarker might help to discern between infection (where PCT will decrease) and poor outcome (where PCT will not decrease). Furthermore, a PCT value under 2 ng/mL may indicate the absence of complications and poor outcomes in the postoperative period. However, further research should be carried out to confirm these results.

## Supporting information

S1 File (RAR)
